# Permissive State of EMT: The Role of Immune Cell Compartment

**DOI:** 10.3389/fonc.2020.00587

**Published:** 2020-04-22

**Authors:** Vita Fedele, Davide Melisi

**Affiliations:** Digestive Molecular Clinical Oncology Research Unit, Section of Medical Oncology, Università degli Studi di Verona, Verona, Italy

**Keywords:** EMT, cytotoxic T cells, Treg, inflammatory cytokines, TGF-β

## Abstract

The Epithelial to Mesenchymal Transition (EMT) type 3 is a reversible dynamic process recognized as a major determinant of the metastatic event, although many questions regarding its role throughout this process remain unanswered. The ability of cancer cells to migrate and colonize distant organs is a key aspect of tumor progression and evolution, requiring constant tumor cells and tumor microenvironment (TME) changes, as well as constant changes affecting the cross-talk between the two aforementioned compartments. Alterations affecting tumor cells, such as transcription factors, trans-membrane receptors, chromatin remodeling complexes and metabolic pathways, leading to the disappearance of the epithelial phenotype and concomitant gaining of the undifferentiated mesenchymal phenotype are undoubtedly major players of the EMT process. However, several lines of evidence point out toward a more critical role of TME composition in creating an “EMT-permissive state.” The “EMT-permissive state” consists in changes affecting physical and biochemical properties (i.e., stiffness and/or hypoxia) as well as changes of the TME cellular component (i.e., immune-cell, blood vessel, lymphatic vessels, fibroblasts, and fat cells) that favor and induce the epithelial mesenchymal transition. In this mini review, we will discuss the role of the tumor microenvironment cellular component that are involved in supporting the EMT, with particular emphasis on the immune-inflammatory cells component.

## Introduction

The importance of the tumor microenvironment in relation to tumor progression and metastatization was first highlighted in 1889 by S. Paget when he proposed the “seed and soil theory” to explain the process of distant organs colonization by the primary tumors (1). Although the theory was formulated to explain the preferential migration of certain tumors toward specific organs, it has given the foundation for a better characterization of tumor microenvironment (TME), including its interaction with tumor cells, its tumor grow promoting role and its role in chemotherapy response. At the primary site the TME components play a crucial role in sustaining and facilitating tumor transformation and growth; at the metastatic site TME components play a crucial role in creating a favorable environment for the tumor cells, allowing them to re-initiate tumor growth and establish a new tumor. In both cases normal components of the TME undergo to a series of biochemical and molecular changes creating a “permissive state” sustaining tumor survival (2).

In the following sections, we will highlight some of the changes affecting the TME that occurs during EMT progression, mainly addressing the immune-inflammatory cells component. We will give a brief review of the interplay between the cellular components of the adaptive immune response (T helper cells, also known as CD4+ T cells and cytotoxic T cells, also known as CD8+ T cells) during tumor progression. In particular, we will illustrate how at the tumor site CD4+ and CD8+ T cells, via production of key inflammatory cytokines, exert an active role in supporting inflammation and mediating tumor progression and escape.

## TME Inflammatory Components and Their Relevance in EMT

Defined by Hanahan and Weinberg, in their revised hallmark of cancer (3), as one of the two enabling characteristics of cancer, inflammation can contribute to tumor growth and progression by supplying the TME with bioactive molecules that induce EMT, among other changes. *In vitro* studies conducted on epithelial cancer cells have shown that when cancer cell lines of different origin are incubated with either supernatants derived from a mixed lymphocyte population, or a mix of inflammatory cytokines (TGF-β, IFN-γ, TNF-α) they undergo through a series of changes typical of the EMT, namely remarkable enhancement of *snail1* and *snail2* genes transcription and down-regulation of E-cadherin expression, accompanied by an up-regulation of Vimentin. This and other studies have proven that inflammation is a process tightly associated with the EMT process (4, 5). The tight relationship between inflammation and EMT has been extensively revised elsewhere (6–11). For instance, it has been well-established that several inflammatory cytokines are the main culprits in inducing EMT: IL-6, produced by tumor cells, TAMs, and T cells, via the activation of the IL-6/STAT3 pathway; IL-8, produced by cancer cells, endothelial cells, and CAF, acting in a paracrine and autocrine manner facilitates the acquisition of a mesenchymal phenotype; TNF-α, produced by cells of the innate immune system, including activated macrophages, T lymphocytes, and NK cells, via induction of stemness in cancer cells (12); and, most importantly, TGF-β, via up-regulation of Snail transcription factor in macrophages and induction of their polarization toward an M2-like phenotype (13).

Besides stromal cells (such as mesenchymal and fibroblasts), cells of the innate immune system (natural killer cells, neutrophils, myeloid-derived suppressor cells, dendritic cells, mast cells, and macrophages) as well as cells of the adaptive immune cells (T and B lymphocytes) populate the TME. Several studies performed in different type of tumors, including breast, ovarian, pancreatic, melanoma, and hepatocellular carcinomas have shown that as the EMT progress a shift from an immune profile enriched with neutrophil cells to an immune profile enriched with macrophages is observed. Gene expression studies have shown that in tumor with a mesenchymal signature (enriched with EMT-related gene signature) a decrease in the number of tumor infiltrating lymphocytes (TILs), accompanied by an increased expression of immunosuppressive cytokines (e.g., TGF-β and IL-10) and inhibitory immune check point molecules (CTL4, T-cell immunoglobulin, and TIM-3) is often observed (14). Additionally, a critical study performed on just under 2000 different tumors highlighted a strong correlation between EMT and markers identifying inhibited or exhausted immune responses (15).

These observations suggest that these changes of the TME composition, consisting in a reduced infiltration of immune cells and presence of suppressive or exhausted immune cells, might be required or facilitate the process of EMT. It is, indeed, generally recognized the pivotal role of an altered innate and adaptive immune response in enhancing tumor growth via selection of aggressive clones, induction of immunosuppression, and stimulation of cancer cell proliferation and metastasis (16).

## CD4 and CD8 T Cells, Player of the Adaptive Immune Response

Originating from a common lymphoid progenitor cell, the T helper (Th) CD4+ cells and the cytotoxic (Tc) CD8+ cells are two main key players mediating the adaptive immune response (17) and, together with TAMs, are the most abundant cell type present in the TME of several different types of solid tumors (18). After differentiation in the thymus cortex T cells acquire the ability to recognize either class II MHC or class I MHC; but only after exposure to antigens both CD4+ and CD8+ T cells differentiate into committed distinct subgroups of cells. Depending on the type of cytokines released at site of activation, Th cells can differentiate into Th1, Th2, and Th17 (19, 20). Similarly, CD8+ T cells also can differentiate into T cytotoxic cell type 1 (Tc1) and T cytotoxic cell type 2 (Tc2) upon type 1 or type 2 cytokine responses (20). Cytokines responsible for the above mentioned differentiation processes, are released by both tumor cells and other immune cells (i.e., TGF-β and IL-6).

The presence of tumor-specific CD4 and CD8 T cells at the tumor site is in many cases considered a good prognostic factor (21, 22); however studies conducted in different type of cancers over the past decade have shown that, whereas the infiltration by CD8 T cells is generally considered sign of a good prognosis, the ratio CD8 T cells/Tregs has a more critical role as prognostic factor (23). A higher ratio CD8 T cells/Tregs has been found to be predictive of a favorable outcome in ovarian and liver cancer (24, 25). On the other hand, a lower ratio CD8 T cells/Tregs has been reported to be a predictive factor of poor prognosis in certain type of cancers, such as gastric and breast (26, 27) and predictive of better prognosis for other types of cancers [i.e., lymphomas and colorectal; (28, 29)]. Unfortunately, the mechanism behind this opposing role of Tregs is not fully understood.

Upon delivery of antigens (in the case of cancer, represented by cancer cells antigens) to the lymph nodes and subsequent processing by APC, T cells are activated and migrate to the TME. CD8 T cells evolve into cytotoxic T lymphocytes (CTLs) and exert their anti-tumoral activity, resulting in destruction of tumor cells (30, 31). The anti-tumoral response is supported by CD4 T helper 1 cells (Th-1) via secretion of pro-inflammatory cytokines (i.e., IFN-γ, TNF-α, and IL-2), inducing T-cell activation and CTL cytotoxicity, as well as the anti-tumoral activity of NK cells and macrophages (32, 33). The scenario during the early stages of tumorigenesis consists in recognition and destruction of cancer cells expressing highly immunogenic antigens by CTLs (31), and escape of the less immunogenic tumor cells, which acquire an immune-resistant phenotype.

In the course of cancer progression tumor cells advance developing mechanisms that, on one hand, mirror peripheral tolerance (preventing the local cytotoxicity response of effector T cells), on the other, produce new antigens that impair the immune system priming activity of new range of T cells, therefore, altering the efficacy of tumor control. As tumors keep progressing the recruitment of the second major T cell population, CD4 T cells (Tregs) takes place. The role of Tregs consists in repressing the development and activation of other effector immune cells, such as Th1, CTLs, macrophages, NK, and neutrophils, as well as their cytotoxicity (34). There are several mechanism of action by which Treg cells control the immune response: inhibition by immunoregulatory cytokines (i.e., IL-10, IL-35, and TGF-β); inhibition by effector cells cytolysis, via production of perforin and granzyme; metabolic pathways disruption; interaction with dendritic cells that modulates their function and maturation (contact-dependent mechanism) (35, 36). The contact-dependent mechanism employed by Treg in mediating the immunosuppression consists in the expression of CD39/73, LAG-3, PDL-1, and CTLA4 or PD1 (37–39). Tregs can interact with DCs (CD80 and CD86) through CTLA-4. Upon this interaction DCs produce and release several metabolites like INF-γ, a strong inducer of Indoleamine 2,3-dioxygenase (IDO), enzyme that catalyzes the conversion of tryptophan into a metabolite with immunoregulatory activity (40, 41). IDO degrades tryptophan through different pathways with the production of anthranilic acid, kynurenic acid, 3-hydroxyanthranilic acid, quinolinate and 3-hydroxykynurenine (41). Due to the reduction of tryptophan there is an increase of tRNA without amino acid load, resulting in the activation of General Control Non-derepressible Kinase 2 (GCN2), which hyperphosphorylates the Translation Initiation Factor (eIF2) causing protein synthesis suppression. The formation of the eIF2, GTP, and the Met-RNAi (initiation transfer RNA for methionine) complex to the 40S ribosome is therefore impaired resulting in arrest in G1 phase of the cell cycle and “anergy” of effector T cells. In addition, metabolites derived from tryptophan degradation (quinolinic acid and 3-hydroxyanthranilic acid) have been shown to promote apoptosis in Th1 cells by caspase-8 activation (42, 43). Notably, the 3-hydroxyanthranilic acid metabolite has also been reported to promote activated T cells dead by glutathione depletion (44).

The contact-independent mechanism by which Treg cells control the immune response consist in the production of immunoregulatory cytokines such as IL-10, TGF-β, and IL-35 that have been reported to be correlated with mesenchymal tumor type (45).

Besides Tr1 cells, IL-10 is produced by other cell types consisting of neutrophils, dendritic cells, monocytes, other T lymphocytes, and B lymphocytes (46). Its main function consists in the inhibition of inflammatory cytokines production such as IL-12 (which in turn cause a decrease of Th1 response and INF-γ production) and induction of phagocytic activity. IL-10 exerts its function via JAK1 and TYK2 activation (proteins associated to STAT1, STAT3) (47). The binding of IL-10 to its receptor on Tr1 cells induces JAK1 and TYK2 phosphorylation and activation of STAT3. Subsequently, STAT3 translocates into the nucleus were it promotes SOCS3 transcription, which in turn, inhibits the NF-κB-induced factor MYD88, causing inhibition of the IL-1β, IL-6, and TNF-α (48). IL10-10 exerts its effect on STAT3 also by induction of translocation and phosphorylation to the nucleus in Treg cells, causing more IL-10 production (49). Notably, several studies have demonstrated that STAT-3 activation in tumor cells and tumor-associated immune cells promotes cancer metastasis via EMT (50).

TGF-β is the central inflammatory cytokine in TME and its role in mediating EMT in different types of cancer has been extensively studied and well-established (51–57). It is known that TGF-β can have a dual role in cancer progression: it can exert an anticancer role by suppressing tumor proliferation, inducing apoptosis and cell differentiation, but it can also facilitate cancer development and metastasis (58).

TGF-β signaling inhibition, through impairment of both canonical Smad-dependent and non-canonical Smad-independent signaling, has been proposed as a sound strategy for the treatment of pancreatic cancer patients in combination with classic chemotherapeutic agents or immune checkpoint inhibitors (59–61). Moreover, several studies have demonstrated the critical role of the serine/threonine kinase TGFβ- activated kinase 1 (TAK1) (62) in sustaining resistance to chemo and radiotherapy in pancreatic (63) and esophageal carcinoma (64), by integrating signals from various cytokines and controlling, in turn, the activation of different transcription factors, including NF-κB (65, 66).

TGFβ exerts its effect not exclusively on epithelial cancer cells but also on several immune cell types throughout a combination of mechanisms including suppression of effector T cell differentiation; induction of naïve T cells differentiation into regulatory T or Th17 cells; inhibition of B cells and T cells proliferation; and inhibition of macrophages, dendritic cells, and NK activity (67). The dissociation of TGFβ from LAP (Latency-Associated Peptide) is required for the activation of the signal. While Tregs have a high LAP/TGF-β expression (inhibitory activity) monocytes and dendritic cells have a high TGF-β receptors expression, enabling cell-to-cell interaction and activation. Dendritic cells maturation is also under TGF-β control via induction of IDO, an enzyme that inhibits T cell proliferation. Via induction of FOXP3, TGF-β supports, and facilitates T naïve cell differentiation into Treg cells via induction of FOXP3 expression; whereas, in the presence of IL-6 it promotes differentiation into Th17.

Treg cells (Tr35) produce the immunosuppressor Interleukin 35 (IL-35), although other cells populating the TME are capable of producing this cytokine [i.e., TAMs; (68)]. The two main functions of Tr35 consist in suppression of Th proliferation and induction of naïve T cells conversion into highly suppressor Treg cells (iTr35) (69). The transduction of IL-35 signaling, through the STAT1-STAT4 heterodimer, induces the expression of the IL-35 subunits *IL12A* and *EBI3* in the form of a positive feedback loop (70). The role of IL-35 in promoting tumor progression and metastasis has been highlighted in several studies conducted in pancreatic ductal adenocarcinoma and Hepatocellular carcinomas (71, 72). It has been proposed that IL-35 is one of the major players of the EMT/MET process exerting a dual role: at the site of a primary tumor up-regulation of IL12Rβ2, a subunit of the IL-35 receptor, in transformed cells help them responding to IL-35 during metastasis; at the metastatic sites, TAMs-derived interleukin-35 (IL-35) facilitates tumor colonization through JAK2–STAT6-GATA3 signaling activation, inducing Mesenchymal to Epithelial transition (MET) of cancer cells (73).

## Concluding Remarks

Discerning the cross-talk between cancer cells and components of the tumor microenvironment, especially component of an immune-altered microenvironment, are of crucial importance in trying to better understand the mechanism of the Epithelial to Mesenchymal transition. Different subclasses of an altered immune TME compartment might differ in their ability of inducing tumor growth and metastasis; moreover their activity might change during different stages of cancer in order to support the metastatic process of cancer cells. In other words, cellular components of the TME also undergo through EMT and in doing so contribute to the formation of a permissive state of Epithelial to Mesenchymal Transition. Understanding the biology and function of tumor cells infiltrating lymphocytes can give as a better chance of developing more effective cancer treatment, for instance better designed immunotherapeutic drugs.

## Author Contributions

VF and DM contributed to the conception of the manuscript and final draft of the manuscript. VF wrote the first draft and performed revision of the manuscript. DM added sections of the manuscript.

## Conflict of Interest

The authors declare that the research was conducted in the absence of any commercial or financial relationships that could be construed as a potential conflict of interest.

## Abbreviations

TGF-β: transforming growth factor beta
IFN-γ: interferon gamma
TNF-α: tumor necrosis factor alpha
Snail: snail family of zinc-finger transcription factors
IL-6: Interleukin 6
TAMs: tumor associated macrophages
STAT3: signal transducer and activator of transcription 3
IL-8: interleukin 8
CAF: cancer associated fibroblasts
NK: natural killer
IL-10: interleukin 10
CTLA4: cytotoxic T lymphocyte antigen 4
TIM-3: T-cell immunoglobulin mucin-3
CD4: cluster of differentiation 4
CD8: cluster of differentiation 8
MHC: major histocompatibility complex
APC: antigen presenting cell
LAG-3: lymphocyte activation 3
PDL-1: programmed cell death ligand 1
PD1: programmed cell death protein 1
DC: dendritic cell
JAK1: Janus kinase 1
TYK2: tyrosine kinase 2
SOCS3: suppressor of cytokine signaling 3
MYD88: myeloid differentiation primary response gene 88
FOXP3: forkhead box P3
EBI3: Epstein-Barr virus induced 3
JAK2: Janus kinase 2
STAT6: signal transducer and activator of transcription 6.
